# Bilirubin attenuates the renal tubular injury by inhibition of oxidative stress and apoptosis

**DOI:** 10.1186/1471-2369-14-105

**Published:** 2013-05-17

**Authors:** Se Won Oh, Eun Seong Lee, Sejoong Kim, Ki Young Na, Dong Wan Chae, Suhnggwon Kim, Ho Jun Chin

**Affiliations:** 1 Department of Internal Medicine, Eulji General Hospital, Eulji University College of Medicine, Seoul, Korea; 2 Department of Internal Medicine, Seoul National University Bundang Hospital, Kyeong-Kido, Korea; 3 Department of Internal Medicine, Seoul National University, Seoul, Korea; 4 Renal Institute, Seoul National University Medical Research Center, Seoul, Korea

**Keywords:** Apoptosis, Bilirubin, Cyclosporine, Oxidative stress, Renal injury

## Abstract

**Background:**

Bilirubin (BIL) has been recognized as an endogenous antioxidant that shows a protective effect for cardiorenal diseases. We investigated whether administration of BIL had a protective effect on cyclosporine (CsA)-induced nephropathy (CIN), and examined the effects of BIL on the oxidative stress and apoptosis.

**Methods:**

BIL was pretreated intraperitoneally three times for a week (60 mg/kg), and CsA was injected for 4 weeks (15 mg/kg/day, subcutaneous). Proximal tubular epithelial (HK2) cells were pretreated with 0.1mg/ml of BIL for 24 hours, and then treated with 20 μM of CsA for another 24 hours.

**Results:**

CsA induced marked increases in urine kidney injury molecule-1 (Kim-1) and neutrophil gelatinase-associated lipocalin (NGAL) concentrations (*P* < 0.05). BIL reduced urine Kim-1 in CIN (*P* < 0.05), while urine NGAL exhibited a decreasing tendency. In CsA-treated rat kidneys, the protein expression of NOX4 and p22phox was reduced by BIL (*P* < 0.05). BIL ameliorated CsA-induced arteriolopathy, tubulointerstitial fibrosis, tubular injury, and the apoptosis examined by TUNEL assay (*P* < 0.01). In HK2 cells, BIL reduced intracellular reactive oxygen species in CsA-treated cells. CsA increased the protein expression of bax, cleaved caspase-9, caspase-3 and the activity of caspase-3; however, the anti-apoptotic bcl-2 protein was reduced. These changes were recovered by BIL (*P* < 0.05).

**Conclusions:**

The direct administration of BIL protected against CsA-induced tubular injury via inhibition of oxidative stress and apoptosis.

## Background

Bilirubin (BIL) has been shown to exert a protective effect for cardiorenal diseases. A high-normal concentration of serum BIL was related to a decreased risk of cardiovascular disease [1-3]. Also, BIL showed beneficial effects in diabetic nephropathy, ischemia reperfusion injury, and contrast-induced nephropathy [4-6]. BIL is a breakdown product of heme-containing proteins such as hemoglobin in aging red blood cells. Heme oxygenase-1 (HO-1), the rate-limiting enzyme involved in heme catabolism, converts heme to biliverdin, free ferrous iron, and carbon monooxide. Subsequently, biliverdin is rapidly converted to BIL by biliverdin reductase [7]. Recently, accumulating evidence has suggested that HO-1 and its product BIL may be important endogenous agents with cytoprotective activity against oxidative stress injury [5-9].BIL is known as an effective radical scavenger, and inhibits the activity of nicotinamide adenine dinucleotide phosphate (NADPH) oxidase [5,10]. BIL had been showed the antioxidant and cytoprotective effects in angiotensin II-mediated vasoconstriction and DNA damage [11]. However, few studies have reported the anti-apoptotic effect of BIL [6,12,13].

Cyclosporine (CsA) has been commonly used as an immunosuppressant in organ transplantation and auto-immune diseases. However, chronic use of CsA has a toxic effect on the kidney. CsA induces the activation of the intrarenal renin–angiotensin-aldosterone system [14,15], which causes renal vasoconstriction and hypoxia. In CsA-induced nephropathy (CIN), the production of reactive oxygen species (ROS) is increased in the kidney [16,17]. In addition, CsA may activate a NADPH oxidase that releases superoxide anion [18]. CsA activates the expression of pro-apoptotic genes and induces apoptosis in renal tubular and interstitial cells, resulting in tubular atrophy [19-21].

We investigated whether intraperitoneal administration of BIL had a protective effect on CIN. We also examined the molecular mechanism underlying the effects of BIL on the oxidative stress and apoptosis in CIN.

## Methods

### Cell culture

HK-2 cells (ATCC CRL-2190), which are proximal tubular epithelial cells derived from normal human kidney tissue, were cultured using Renal Epithelial Basal Medium (Lonza Walkersville Inc., Walkersville, MD, USA) with recommended supplements included in the REGM Singlequot Bulletkit. The cells were fed two to three times weekly and subcultivated via trypsinization when near confluence. HK-2 cells between *passages 10 and 25* were used for these experiments.

### Cell treatment

Cells were grown to 80% confluence before treatment for all experiments. CsA (Sigma, St. Louis, MO, USA) was prepared as a stock solution (4.2 mM) by dissolving 5 mg of the powder in 1 ml of absolute ethanol. This stock was further diluted in growth medium before treatment and added to the main cell growth media. BIL (Sigma #B4125, St. Louis, MO, USA) was dissolved in Dimethyl sulfoxide.

The cells were divided into three groups: control cells, CsA-treated cells, and CsA-treated cells with BIL pre-treatment (BIL + CsA). On the day prior to an experiment, the cells were pre-treated with 0.1 mg/ml of BIL for 24 hours. The cells were then treated with 20 μM of CsA for another 24 hours. Only bilirubin treated cells (BIL) was evaluated for the expression of caspase-3.

### Animal experiments

Fifteen male Sprague–Dawley rats (Orient Bio Inc., Seongnam, Korea) weighing 200 to 250 g were housed in individual cages in a temperature- and light-controlled environment. The rats received a low-salt diet (0.05% sodium; Samtako, Osan, Korea) and were allowed free access to water. This study was approved by, and conducted according to the guidelines of, the Seoul National University Bundang Hospital Committee of animal experiment. After one week on the low-salt diet, the rats were assigned to experimental groups (N = 5/group). BIL was injected intraperitoneally three times for one week (60 mg/kg) before the administration of CsA. The BIL was dissolved in DMSO to a final concentration of 20 mg/ml. The vehicle consisted of DMSO. CsA (Novartis, East Hanover, NJ, USA) was diluted in olive oil to a final concentration of 15 mg/ml. The following groups were studied: (1) Vehicle (VH) The rats received a subcutaneous (SC) injection of olive oil 1 ml/kg/day plus a intraperitoneal injection of DMSO; (2) Bilirubin (BIL): The rats received a SC injection of olive oil 1 ml/kg/day plus a intraperitoneal injection of bilirubin (60 mg/kg) dissolved with DMSO (3) CsA group: The rats received a SC injection of CsA 15 mg/kg/day plus a intraperitoneal injection of DMSO; (4) BIL + CsA group: The rats received a SC injection of CsA 15 mg/kg/day plus a a intraperitoneal injection of BIL (60 mg/kg) dissolved with DMSO.

After 4 weeks, the rats were anesthetized with zolazepam and xylazine, their blood was sampled, and their kidneys were collected. The left kidney from each rat was fixed in 10% phosphate-buffered formalin for morphologic analyses. The right kidneys were collected for western blotting.

### Physiologic measurements

At the end of experiments, the rats were weighed and placed in metabolic cages, and urine was collected for 24 h. The urine volume was measured, and the albumin concentration was determined by ELISA kit (Exocell, Philadelphia, PA, USA). Urinary albumin excretion was calculated in terms of milligrams of albumin per 24 hours. Creatinine levels in the serum (Bioassay Systems, Hayward, CA, USA) and urine (Exocell, Philadelphia, PA, USA) were also measured using ELISA kit. CsA blood level was measured by a direct CsA radioimmunoassay kit (Immunotech, Czech Republic). Serum LDH was measured by using an automatic analyzer (ADVIA 2400, Siemens, USA). Bilirubin was measured by quantitative colorimetric assay (Bioassay systems #DIBR-180, CA, USA). Systolic blood pressure (SBP) was monitored with a tail cuff sphygmomanometer (Panlab S.L., Barcelona, Spain). SBP was recorded as the mean value of three separate measurements that were obtained at each session.

### The measurement of kidney injury molecule-1 (Kim-1) and neutrophil gelatinase-associated lipocalin (NGAL) in urine

The urine samples were centrifuged immediately after being collected, and the supernatant was preserved at -70°C until use. The Kim-1 and NGAL were examined with the accompanying procedural instructions by using a Rat Kim-1 ELISA Kit (Bioassay Works LLC, Ijamsville, MD, USA) and a Rat NGAL ELISA Kit (BioPorto Diagnosis A/S, Denmark) in Sandwich ELISA analysis, respectively. The frozen and preserved samples were thawed at room temperature. The average values were derived in duplicate for all of the samples. The standard curve and the absorbance of the samples were measured with a micro-plate reader (Bio-Rad Laboratories, Inc., CA, USA) at a wavelength of 450 nm with reference reading at 650 nm.

### Histologic analyses

The tissue samples used for light microscopy and immunoperoxidase staining were fixed in formalin and embedded in paraffin. Three-micrometer sections were stained with periodic acid-Schiff (PAS) or Masson Trichrome (MT). The histologic findings were subdivided into three categories: arteriolopathy, tubulointerstitial fibrosis, and tubular injury. Findings ascribed to tubulointerstitial fibrosis included matrix expansion with tubular distortion and basement membranes thickening. Tubular injury consisted of cellular and intracellular vacuolization, tubular collapse and tubular distension. More than 20 consecutive fields were examined under × 200 magnification and the results were averaged. The extent of tubulointerstitial fibrosis and tubular injury in cortical tubules were graded using the following score: 0 = normal interstitium, 0 = normal interstitium, 0.5 = <5% of areas injured, 1 = 5 to 15%, 1.5 = 16 to 25%, 2 = 26 to 35%, 2.5 = 36 to 45%, and 3 = > 45%. Arteriolopathy was determined by counting at least 100 glomeruli. During this counting, arteriolopathy was recorded as present or absent. Arteriolopathy consisted of the hyalinization and destruction of afferent arterioles. The results are expressed as the percentage of juxtaglomerular affected arterioles over total number of arterioles: 0 = no arterioles injured, 0.5 = <15%; 1 = 15 to 30%, 1.5 = 31 to 45%, 2 = 46 to 60%, 2.5 = 61 to 75%, and 3 = >75%.

### Western blot analysis

Western blotting was performed as previously described [22]. The kidney tissue was homogenized, and the lysates of kidney tissue and HK-2 cell were prepared. Protein concentrations were measured using a bicinchoninic acid protein assay kit (Thermo Fisher Scientific, Rockford, IL, USA). The samples were run on SDS-polyacrylamide mini-gels (Bio-Rad Mini Protean III). The proteins were transferred to nitrocellulose membranes by electroelution. NOX-4 (Santa Cruz Biotech, Santa Cruz, CA), p22phox (Santa Cruz Biotech, Santa Cruz, CA), bcl-2 (Cell Signaling Technology, Beverly, MA), bax (Santa Cruz Biotech, Santa Cruz, CA), caspase-9 (BD Bioscience, Franklin Lakes, NJ), caspase-3 (Cell Signaling Technology, Danvers, MA), β-actin (Santa Cruz Biotech, Santa Cruz, CA), were used for this study. Incubation with horseradish peroxidase-conjugated secondary antibodies (Santa Cruz Biotech, Santa Cruz, CA, USA) was followed by band visualization using an enhanced chemiluminescence substrate (Thermo Fisher Scientific, Rockford, IL, USA). The band densities were quantified by densitometry (GS-700 Imaging Densitometry, Bio-Rad, Hercules, CA, USA). To facilitate comparisons, the densitometry values were normalized by β-actin expression.

### Detection of intracellular reactive oxygen species (ROS)

Oxidation-sensitive 2′,7′-dichlorofluorescein diacetate (DCFH-DA) (Sigma, St. Louis, MO, USA) was used to determine the intracellular production of reactive oxygen species (ROS). The cells were loaded with DCFH-DA at a final concentration of 10 μM, incubated at 37°C for 30 min, washed with phosphate-buffered saline, and removed from the dishes by scraping. The fluorescence intensity was measured by fluorescence spectrophotometer at excitation and emission wavelengths of 490 nm and 526 nm, respectively.

### Detection of apoptosis

Apoptosis was assessed by terminal deoxynucleotidyl transferase-mediated uridine triphosphate nick-end labeling (TUNEL), following which the apoptotic cells were counted. Apoptotic cells were defined by chromatin condensation or nuclear fragmentation. Apoptosis was detected in the specimens using the In Situ Cell Death Detection Fluorescein Kit (Roche Applied Science, Mannheim, Germany) according to the manufacturer’s protocol. The same slides were stained with 4′,6′-diamidino-2-phenyindole (DAPI) in phosphate-buffered saline to reveal total nuclei. For apoptotic nuclei counting, cells from at least 10 consecutive fields under × 400 magnification were counted. The final count was expressed as the percentage of total cells counted by fluorescence microscopy (Carl Zeiss, Jena, Germany). In addition, TUNEL-positive cells were counted in the cortical tubular cells in 10 consecutive fields under × 400 magnification.

Caspase-3 activity assayed through the use of the Caspase-3/CPP32 Fluorometric Assay Kit (BioVision, Mountain View, CO, USA). Cells were incubated in cell lysis buffer and centrifuged at 14,000 rpm, and the supernatants were incubated with DEVD-AFC (a specific substrate for caspase-3) at 37°C for 1 hour. Subsequently, the activity was assayed through the use of a fluorescence microplate reader (Molecular Devices, Sunnyvale, CA, USA).

### Statistical analyses

The results are presented as the mean ± standard deviation of mean. The statistical analyses were performed using SPSS (version 18.0. for Windows; SPSS Inc., Chicago, IL, USA). The comparisons between groups were conducted with an analysis of variance followed by a Tukey. For comparisons of two groups, data were analyzed using a Student’s t-test or a Mann–Whitney test. The level of statistical significance was set as *P <* 0.05.

## Results

### Intraperitoneal injection of bilirubin (BIL) significantly increase the plasma BIL

**Figure 1:**
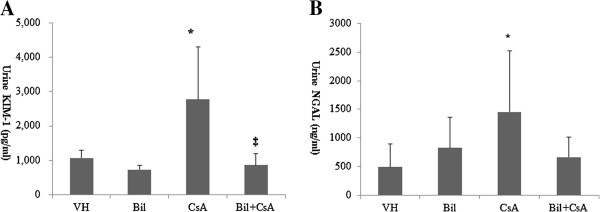
**Intraperitoneal injection of bilirubin (BIL) significantly increase the plasma BIL.** *P < 0.05 vs. 0 hours.

Total plasma bilirubin concentration was meaured after an intraperitoneal injection of bilirubin. After the administration of bilirubin intraperitoneally (60 mg/kg), 30-fold of increase of plasma bilirubin was noted within 2 hours in the rat and this increase was not significant at 24 hrs after injection (basal, 0.01 ± 0.00 mg/dL; 1 hr, 0.34 ± 0.03 mg/dL; 2 hrs, 0.37 ± 0.07 mg/dL; 5 hrs, 0.14 ± 0.03 mg/dL; 24 hrs, 0.06 ± 0.04 mg/dL, P < 0.001) (Figure 1).

### Bilirubin (BIL) reduced the urine concentration of kidney injury molecule-1 (Kim-1) in CsA-induced nephropathy (CIN)

**Figure 2:**
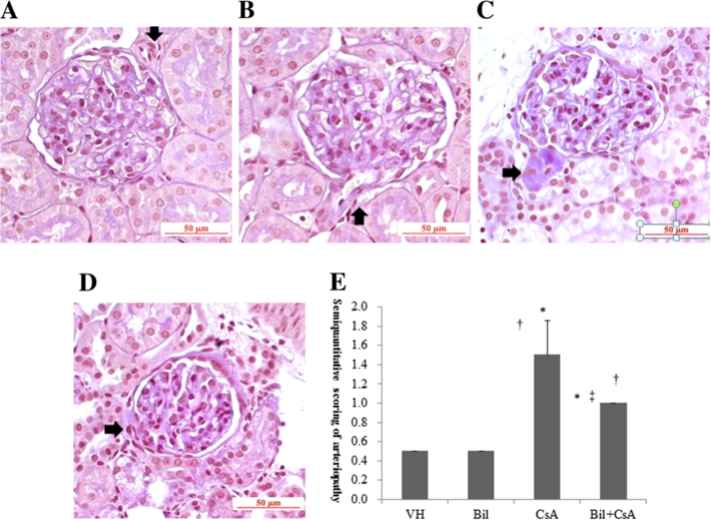
**Urine concentrations of kidney injury molecule-1 (Kim-1) and neutrophil gelatinase-associated lipocalin (NGAL). A**: The urine concentration of Kim-1 is markedly increased in CsA-only treated rats, and is significantly reduced by BIL administration. **B**: The urine concentration of NGAL is markedly increased in CsA-only treated rats, and urine NGAL shows a decreasing tendency in BIL-treated rats. The data are the means ± SD. *P < 0.05 vs. VH, †*P* < 0.05 vs. BIL, and ‡*P* < 0.05 vs. CsA.

Table 1 shows the physiologic parameters of animals at the end of experiment. CsA-treated rats presented a significant increase in serum creatinine (*P* < 0.01), but this was not improved by BIL treatment. The excretion of 24-hr urine albumin was not significantly increased in CsA-treated rats (5.4 ± 4.1 mg/day, 4.8 ± 5.1 mg/day, 9.1 ± 6.5 mg/day, and 6.6 ± 3.0 mg/day in vehicle (VH), BIL, CsA, and BIL + CsA groups, respectively). There were no significant differences in body weight, systolic blood pressure (SBP), lactate dehydrogenase (LDH) , AST, and ALT among the groups. CsA blood level increased in CsA and BIL + CsA groups (P < 0.01). Serum bilirubin was not increased in VH and BIL groups at the time of sacrifice, and increased in CsA and BIL + CsA groups (P < 0.01) (Table 1). Urine NGAL and KIM-1 have been recognized as markers of renal tubular injuries [23,24]. CsA significantly increased the urine concentrations of KIM-1 and NGAL (*P* < 0.05). BIL pretreatment reduced the CsA-induced increase of urine concentration of KIM-1(*P* < 0.05) (urine KIM-1, 1050.0 ± 261.3 pg/ml, 735.0 ± 120.2 pg/ml, 2278.0 ± 1523.3 pg/ml, and 858.0 ± 340.3 pg/ml; VH, BIL, CsA, and BIL + CsA, respectively). In addition, a tendency, although statistically insignificant, for decreased urine NGAL concentration was shown in BIL-pretreated rats; urine NGAL, 512.2 ± 451.3 ng/ml, 852.6 ± 536.3 ng/ml, 1457.5 ± 1060.0 ng/ml and 668.0 ± 345.7 ng/ml; VH, BIL, CsA, and BIL + CsA, respectively (Figure 2A, 2B).

**Table 1:** **Physiologic data **^a,b^

	**VH**	**BIL**	**CsA**	**Bil + CsA**
N	5	5	5	5
Body weight (g)	356.5 ± 29.0	356.1 ± 57.6	318.3 ± 57.6	290.6 ± 10.11
SBP (mmHg)	127.5 ± 14.0	139.8 ± 10.0	149.0 ± 19.8	134.0 ± 15.9
Serum creatinine (mg/dL)	0.7 ± 0.1	0.7 ± 0.2	1.7 ± 0.9*†	1.7 ± 0.3*†
24 hr urine albumin (mg)	5.4 ± 4.1	4.8 ± 5.1	9.1 ± 6.5	6.6 ± 3.0
Serum CsA (μg/mL)	0.02 ± 0.00	0.02 ± 0.00	2.89 ± 0.29*†	3.31 ± 0.40*†
Serum LDH (IU/L)	486.6 ± 183.8	623.4 ± 400.1	748.2 ± 182.6	642.2 ± 193.6
Serum AST (IU/L)	79.7 ± 18.9	86.2 ± 41.1	61.6 ± 6.1	66.0 ± 7.0
Serum ALT (IU/L)	29.3 ± 3.8	34.0 ± 17.4	26.4 ± 3.6	31.0 ± 3.3
Serum bilirubin (mg/dL)	0.03 ± 0.03	0.01 ± 0.02	0.16 ± 0.04*†	00.20 ± 0.06*†

^a^Vehicle (VH): The rats received a subcutaneous (SC) injection of olive oil 1 ml/kg/day plus a intraperitoneal injection of DMSO; Bilirubin (BIL): The rats received a SC injection of olive oil 1 ml/kg/day plus a intraperitoneal injection of bilirubin (60 mg/kg) dissolved with DMSO; cyclosporine (CsA): The rats received a SC injection of CsA 15 mg/kg/day plus a intraperitoneal injection of DMSO; BIL + CsA: The rats received a SC injection of CsA 15 mg/kg/day plus a a intraperitoneal injection of bilirubin (60 mg/kg) dissolved with DMSO; SBP, systolic blood pressure, LDH, lactate dehydrogenase.

^b^Data are displayed as means ± SD.

*P < 0.05 vs. VH, †*P* < 0.05 vs. Bil, and ‡*P* < 0.05 vs. CsA.

### Bilirubin (BIL) ameliorated CsA-induced arteriolopathy and tubulopathy

**Figure 3:**
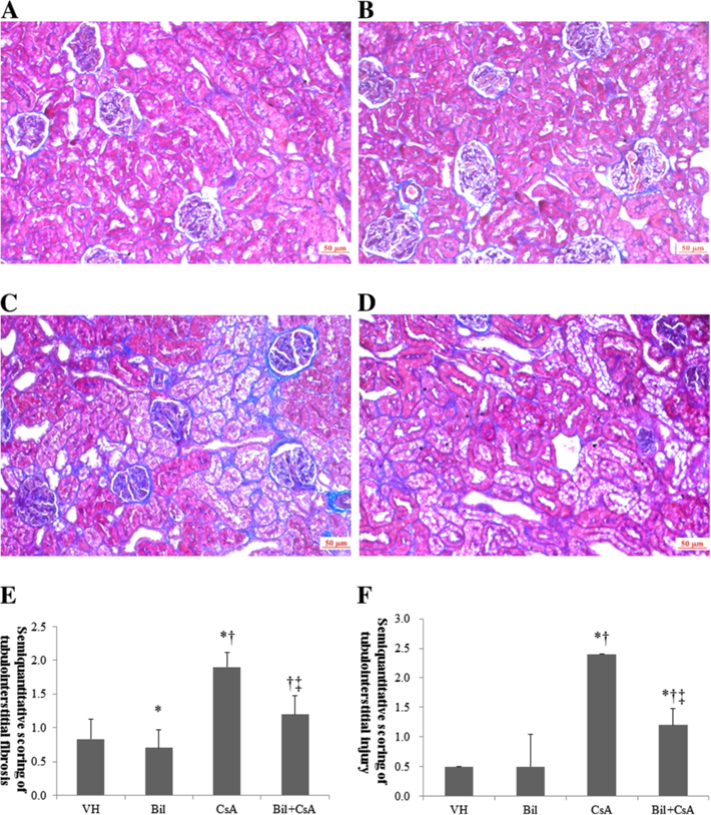
**Afferent arteriolopathy in CsA-treated rat kidneys. A**: Vehicle (VH), **B**: Bilirubin (BIL) **C**: CsA, and **D**: BIL + CsA groups **E**: Semiquantitative scoring of arteriolopathy according to mean percentage of afferent arterioles injured, which was estimated in at least 100 preglomerular afferent arterioles from the **A**, **B**, **C** and **D** groups (PAS stain, ×200). Compared with CsA group, BIL + CsA group displayed afferent arteriolopathy. The data are the means ± SD. *P < 0.05 vs. VH, †*P* < 0.05 vs. BIL, and ‡*P* < 0.05 vs. CsA.

**Figure 4:**
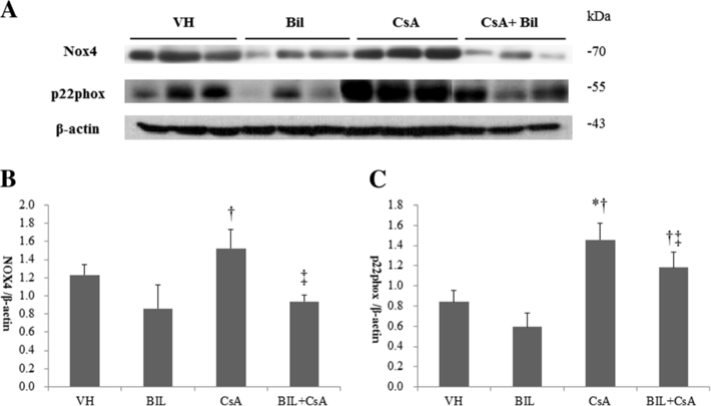
**Tubulointerstitial fibrosis in CsA-treated rat kidneys. A**: Vehicle (VH), **B**: Bilirubin (BIL) **C**: CsA, and **D**: BIL + CsA groups (MT staining, magnification × 200). Semiquantitative scoring of **E**: tubulointerstitial fibrosis and **F**: tubular injury according to mean percentage of injured area obtained in at least 20 fields from the **A**, **B**, **C** and **D** groups. Compared with CsA group, BIL + CsA group showed improved tubulointerstitial fibrosis. The data are the means ± SD. *P < 0.05 vs. VH, †*P* < 0.05 vs. BIL, and ‡*P* < 0.05 vs. CsA.

BIL treatment ameliorated the histopathologic findings in CIN. CsA-only treated animals developed an increase of arteriolar hyalinosis, extensive tubulointerstitial fibrosis and tubular injury (*P* < 0.01) (Figures 3B, 4B). BIL pretreatment with CsA significantly improved the afferent arteriolopathy, tubulointerstitial fibrosis, and tubular injury compared to the CsA-only treated rats (*P* < 0.01) (Figures 3C, 4C). The semiquantitative scores for arteriolar hyalinosis were 0.5 ± 0.0, 0.5 ± 0.0, 1.5 ± 0.4, and 1.0 ± 0.0, in VH, BIL, CsA, and BIL + CsA groups, respectively (Figure 3D). The scores of tubulointerstitial fibrosis were 0.8 ± 0.3, 0.7 ± 0.3, 1.9 ± 0.2, and 1.2 ± 0.3, and of tubular injury were 0.5 ± 0.0, 0.5 ± 0.0, 2.4 ± 0.5, and 1.2 ± 0.3, in VH, BIL, CsA, and BIL + CsA groups, respectively (Figure 4D, 4E).

### Bilirubin (BIL) reduced CsA-induced reactive oxygen species (ROS) in rat kidneys and proximal renal tubular cells

**Figure 5:**
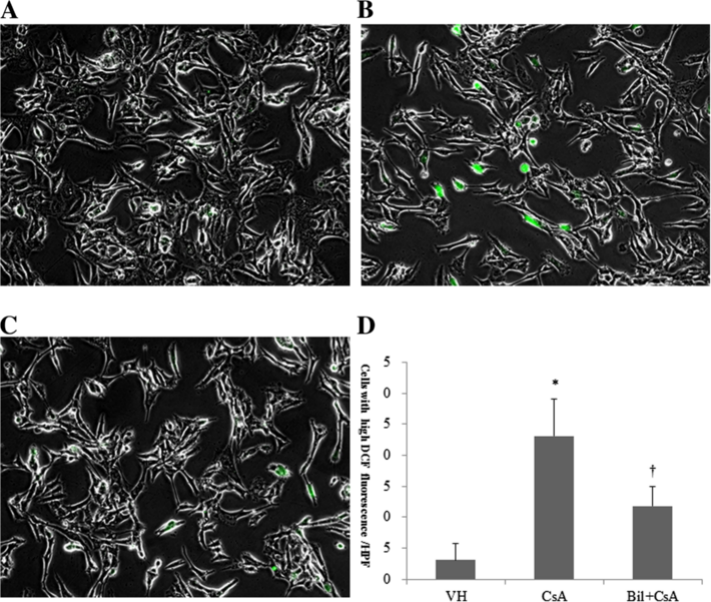
**Bilirubin (BIL) suppresses CsA-induced increased expression of Nox4 and p22**^**phox **^**in rat kidneys. A**: Western blot analysis of Nox4 and p22^phox^ molecules from VH, BIL, CsA, and BIL + CsA groups. **B**: The average densitometric ratio was calculated relative to β-actin for **C**: The average densitometric ratio was calculated relative to β-actin for p22phox. The data are the means ± SD. *P < 0.05 vs. VH, †*P* < 0.05 vs. BIL, and ‡*P* < 0.05 vs. CsA.

**Figure 6:**
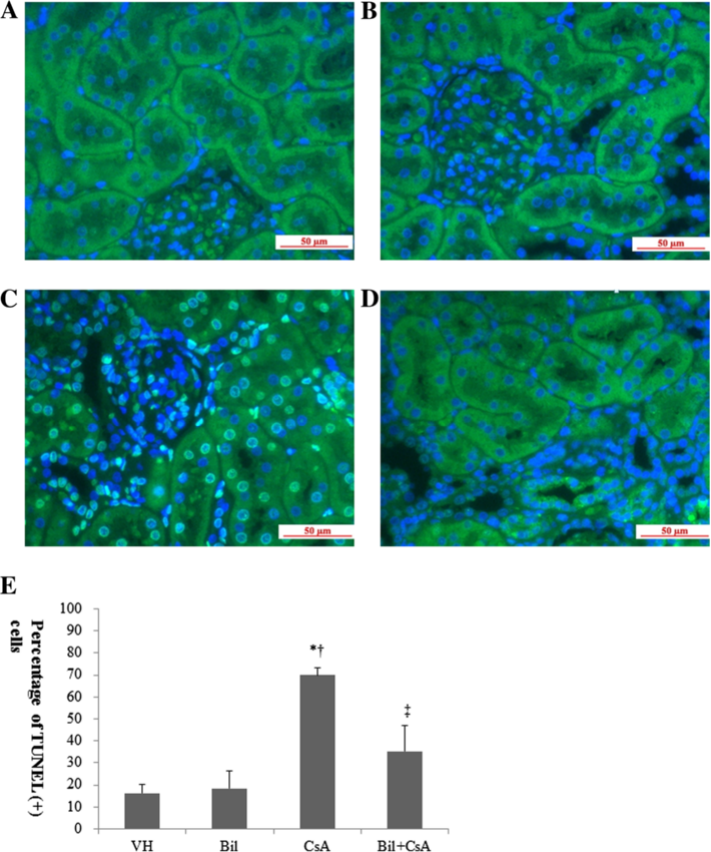
**Bilirubin (BIL) ameliorates intracellular reactive oxygen species (ROS) in CsA-induced nephropathy (CIN).** 2′,7′-Dichlorofluorescein diacetate (DCFH-DA) was used to determine the intracellular production of ROS. **A**: Vehicle (VH), **B**: CsA, and **C**: BIL + CsA groups (magnification × 400). **D**: The number of cells with intracellular ROS obtained in at least 10 fields from the **A**, **B** and **C** groups. The data are the means ± SD. *P < 0.05 vs. control, †*P* < 0.05 vs. CsA.

We determined the activation of NADPH oxidase in response to CsA and BIL + CsA treatment in rat kidneys. CsA treatment increased the expression of NOX4 by western blotting, although statistically insignificant. BIL pretreatment markedly reduced the protein level of NOX4 compared to that of CsA-only treated rats (*P* < 0.05) (Figure 5A, 5B). The expression of p22phox was significantly increased by approximately five fold in CsA-only treated rats (*P* < 0.05), and BIL pretreatment improved the expression of p22phox (*P* < 0.05) (Figure 5A, 5C). We further examined the effect of BIL in the generation of ROS. CsA increased the generation of intracellular ROS revealed by fluorescence of 2′,7′-dichlorofluorescein in HK-2 cells (Figure 6B). However, BIL significantly reduced the production of intracellular ROS (Figure 6C) (*P* < 0.01). The numbers of cells with intracellular ROS were 3.1 ± 2.7 cells/HPF, 23.1 ± 6.0 cells/HPF, and 11.8 ± 3.2 cells/HPF (Figure 6D).

### BIL improved CsA-induced apoptosis in rat kidney sections and proximal renal tubular cells

**Figure 7:**
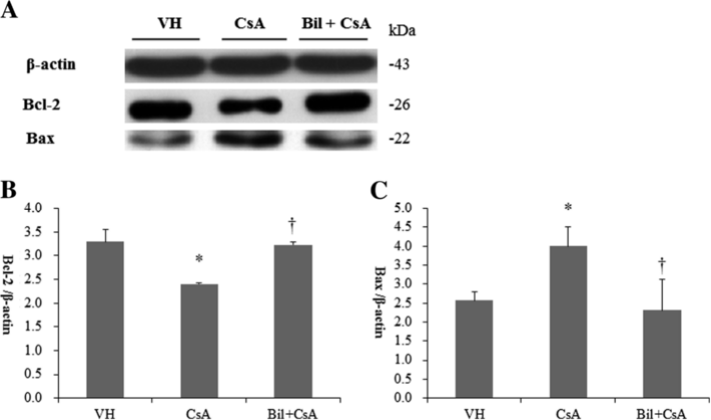
**Bilirubin (BIL) ameliorates apoptosis in CsA-induced nephropathy (CIN).** Representative TUNEL staining of the renal cortex in CIN. **A**: Vehicle (VH), **B**: Bilirubin (BIL) **C**: CsA, and **D**: BIL + CsA groups (magnification × 400). **E**: The percentage of apoptotic cells (TUNEL-positive cells) was obtained in at least 10 fields from the **A***,***B** and **C** groups. The data are the means ± SD. *P < 0.05 vs. VH, †*P* < 0.05 vs. BIL, and ‡*P* < 0.05 vs. CsA.

**Figure 8:**
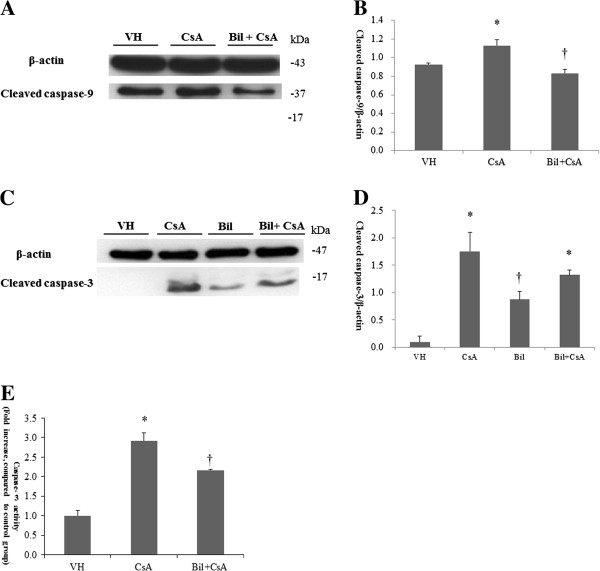
**Bilirubin (BIL) ameliorates apoptosis in CsA-treated HK-2 cells. A**: Western blot analysis of bcl-2 and bax molecules from VH, CsA, and BIL + CsA groups in HK2 cells. **B**: The average densitometric ratio was calculated relative to β-actin for bcl-2. **C**: The average densitometric ratio was calculated relative to β-actin for bax. The data are the means ± SD. *P < 0.05 vs. control, †*P* < 0.05 vs. CsA.

**Figure 9:**
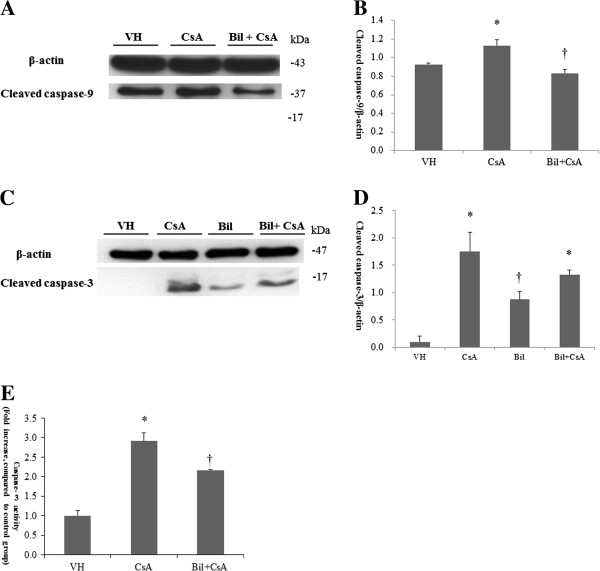
**Bilirubin (BIL) suppresses CsA-induced apoptosis in HK-2 cells. A**: The protein expression of cleaved caspase-9 and cleaved caspse-3 from VH, CsA, BIL, and BIL+CsA groups in HK2 cells. **B**: The average densitometric ratio was calculated relative to β-actin for cleaved caspase-9. **C**: The protein expression of cleaved caspse-3 from VH, CsA, BIL, and BIL+CsA groups in HK2 cells. **D**: The average densitometric ratio was calculated relative to β-actin for cleaved caspase-3. **E**: Caspase-3 activity was determined by caspase-3 fluorometric assay in HK2 cells. The data are the means ± SD. *P<0.05 vs. control, †P<0.05 vs. CsA.

To determine the CSA-induced apoptosis in rat kidneys, we investigated the kidney sections after having detected DNA fragmentations with *in situ* TUNEL assay. In CsA-only treated rats, the apoptotic cells were markedly increased by the administration of CsA in the rat kidneys (Figure 7B). A significantly lower number of apoptotic cells was observed in the BIL-treated rat kidneys (Figure 7C). The percentages of apoptotic cells were 69.9 ± 3.3 and 37.1 ± 11.7 in CsA and BIL + CsA groups, respectively (*P* < 0.01, Figure 7D). The CsA-induced apoptosis appears to be associated with the intrinsic pathway involving intracellular organelles. Therefore, we investigated the intrinsic pathway of apoptosis in CsA-treated human proximal tubular cells. The expression of anti-apoptotic bcl-2 and pro-apoptotic bax was examined by western blotting (Figure 8A). The administration of CsA significantly reduced the abundance of bcl-2 and increased that of bax (*P* < 0.05). These changes were recovered in BIL-pretreated HK2 cells (Figure 8B, 8C). The pro-apoptotic caspase-9 and caspase-3 were also examined by western blotting (Figure 9A). In CsA-only treated cells, the abundance of cleaved caspase-9 was significantly increased. In contrast, BIL pretreatment significantly reduced the abundance of cleaved caspase-9 (Figure 9B) (*P* < 0.05). BIL-only treated group was examined for cleaved caspase-3, and it did not increase the protein expression of cleaved caspase-3 significantly. CsA increase the expression of cleaved caspase-3, and BIL treatment reduced that of cleaved caspase-3 (Figure 9C). Caspase-3 activity was significantly increased in CsA-only treated HK2 cells. BIL pretreatment markedly reduced the activity of caspase-3 in CsA-treated HK-2 cells (P < 0.05, Figure 9D).

## Discussion

In this study, the protective effect of BIL on CIN was examined through the inhibition of oxidative stress and apoptosis. The expression of NADPH oxidase subunits and intracellular ROS was markedly reduced by BIL administration in CsA-treated rat kidneys and HK2 cells, respectively. BIL ameliorated CsA-induced apoptosis by up-regulating anti-apoptotic protein bcl2 and down-regulating pro-apoptotic bax, caspase-9, and caspase-3.

The production of ROS, mainly in the form of superoxide and hydrogen peroxide, has an important role in the initiation and progression of cardiovascular and renal diseases [25,26]. The superoxide is generated by univalent reduction of molecular oxygen, mediated by various enzyme systems such as NADPH oxidases of the Nox family. Two molecules of superoxide react to form hydrogen peroxide accelerated by the superoxide dismutase [27]. CsA induced the production of superoxide ion and hydrogen peroxide in kidneys [16,17]. In the present study, the oxidation of DCFH-DA to fluorescent dichlorofluorescein (DCF) by ROS was significantly increased in CsA-only treated HK2 cells [28]. BIL administration effectively reduced intracellular ROS in CsA-treated HK2 cells. The NADPH oxidases have been recently identified as crucial mediators of renal injury [5,29-31]. Early studies of NADPH oxidases were performed in phagocytic cells. The NADPH oxidases on phagocyte are composed of two plasma membrane-associated proteins, gp91^phox^ and p22^phox^, which form flavocytochrome b_558_, and cytosolic subunits, p40^phox^, p47^phox^, p67^phox^, and the small GTPase Rac1/2. Nox proteins had been found as homologs of gp91^phox^ in renal cells [28]. The most highly expressed Nox homolog in cardiorenal system is Nox4 [31,32], which is abundant in renal tubule, fibroblast, and glomerular mesangial cells [33-35]. Increasing evidence suggests that Nox 4 heterodimerization with p22^phox^ is sufficient to activate the enzyme without any cytosolic factors, contrary to other Nox isoforms [32,36]. In addition, the overall output of ROS may be directly related to the expression level of Nox4 [32]. We confirmed that Nox4 and its docking subunit p22^phox^ are increased by administration of CsA. CsA increased Nox2 mRNA and proteins in rat tubular epithelial cells, and Nox-2 and p22phox were increased in tubulointersitial injury in human allografts [37,38]. However, BIL reduced the protein expression of Nox4 and p22^phox^ in CsA induced renal injury.

The production of oxidative stress may play a principal role in the process of tubular injury [39,40]. HO-1 is the rate-limiting enzyme of heme degradation, and heme is converted to CO and bilirubin by HO-1. HO-1 had showed antioxidant, anti-inflammatory, and cytoprotective effect. HO-1 induction by heme arginate and the treatment of bilirubin attenuate TNF-alpha mediated VCAM-1 production, however, CO did not show the beneficial effect. This data suggest that the anti-atherogenic effect of HO-1 is mediated predominantly by the action of bilirubin [41]. HO-1 is expressed at low levels within the normal kidney and the HO-1 is induced in response to tubulointerstitial injury [42]. In addition, exogenous bilirubin treatment resulted in improvements in renal vascular resistance, glomerular filtration rate, tubular function, and urine output after ischemia-reperfusion injury. Exogenous bilirubin accumulated within the hepatoblastoma HepG2 cells [43].

The apoptosis in renal tubular cells causes tubular dilatation and atrophy in renal injury [44]. Apoptosis is initiated by two distinct pathways: an intrinsic pathway involving mitochondria and an extrinsic pathway leading to the rapid recruitment of Fas-Associated protein with Death Domain and caspase-8 [45]. The apoptosis induced by CsA may be developed through the intrinsic pathway, because it promotes Bax aggregation and translocation to the mitochondria, inducing permeabilization of the outer mitochondrial membrane and release of cytochrome C [45]. In addition, CsA causes a caspase-dependent loss of mitochondrial membrane potential, and activation of caspase-9 and caspase-3 [45,46]. In this study, CsA increased the number of apoptotic cells in rat kidneys, and consequently increased the expression of bax, cleaved caspase-9, cleaved caspase-3 and the activity of caspase-3. In addition, the anti-apoptotic bcl-2 protein, which sequesters Bax and inhibits the activation of caspase-9, was reduced by CsA treatment. However, these alterations were recovered by BIL treatment in CIN.

Kim-1 and NGAL have been proposed as kidney injury markers [23,24]. Kim-1 is a type 1 transmembrane glycoprotein with an immunoglobulin and mucin domain, and NGAL is a protein of the lipocalin family consisting of 8β-strands that form a β-barrel enclosing calyx [23,24]. After renal injury, they are highly up-regulated in damaged renal proximal tubular cells, and can be detected in urine. The degree of renal injury is correlated with the increased urine concentration of NGAL and Kim-1 [23,24,47,48]. The functions of Kim-1 and NGAL are unclear, but their relations with apoptosis have been documented [48-50]. According to the severity of renal damage, both the number of apoptotic cells and the expression of Kim-1 were increased, and Kim-1 was expressed in all the tubules displaying apoptotic labeling [47]. In addition, the up-regulation of Kim-1 and NGAL was identified in CsA-induced renal injury, suggesting that renal tubular cells are injured by CsA [51,52]. We confirmed the reno-protective effect of BIL by the significant reduction of urine Kim-1 and the decreased tendency of urine NGAL concentration, indicating that BIL helps to prevent epithelial cell injury. Histologic improvements in arteriolopathy, tubulointerstitial fibrosis, and tubular injury were found in BIL-treated rat kidneys, and these recoveries appeared to be associated with the reduction of oxidative stress, apoptosis, and tubular damage. The administration of bilirubin could be a target for protecting against the progression of renal injury, but further studies will be needed to facilitate such a potential therapy.

## Conclusions

We showed that the direct administration of BIL protected against CsA-induced tubular injury via inhibition of oxidative stress and apoptosis. BIL may be a protective agent against renal tubular injury, but further studies are needed to develop this potential therapy.

## Competing interests

The authors declare that they have no competing interests.

## Authors’ contribution

SWO and ESL performed the experiments, SWO and HJC analyzed and interpreted the data, SWO and ESL prepared the figures for publication, SWO drafted the manuscript, HJC, SK, KYN, DW C, and SK revised the manuscript, all authors approved the final version of the manuscript, HJC conceived and designed the study.

## Pre-publication history

The pre-publication history for this paper can be accessed here:

http://www.biomedcentral.com/1471-2369/14/105/prepub
